# Changing behaviour ‘more or less’—do theories of behaviour inform strategies for implementation and de-implementation? A critical interpretive synthesis

**DOI:** 10.1186/s13012-018-0826-6

**Published:** 2018-10-29

**Authors:** Andrea M. Patey, Catherine S. Hurt, Jeremy M. Grimshaw, Jill J. Francis

**Affiliations:** 1 School of Health Sciences, City, University of London, 10 Northampton Square, London, EC1V 0HB UK; 2 Centre for Implementation Research, Ottawa Hospital Research Institute – General Campus, 501 Smyth Road, Ottawa, Ontario K1H 8L6 Canada; 3 Faculty of Medicine, University of Ottawa, Roger Guindon Hall, 451 Smyth Road, Ottawa, Ontario K1H 8M5 Canada

**Keywords:** Implementation, De-implementation, Behavioural theory and model, Behaviour change, Health professional, Intervention, Implementation research, Critical interpretive synthesis

## Abstract

**Background:**

Implementing evidence-based care requires healthcare practitioners to do less of some things (de-implementation) and more of others (implementation). Variations in effectiveness of behaviour change interventions may result from failure to consider a distinction between approaches by which behaviour increases and decreases in frequency. The distinction is not well represented in methods for designing interventions. This review aimed to identify whether there is a theoretical rationale to support this distinction.

**Methods:**

Using Critical Interpretative Synthesis, this conceptual review included papers from a broad range of fields (biology, psychology, education, business) likely to report approaches for increasing or decreasing behaviour. Articles were identified from databases using search terms related to theory and behaviour change. Articles reporting changes in frequency of behaviour and explicit use of theory were included. Data extracted were direction of behaviour change, how theory was operationalised, and theory-based recommendations for behaviour change. Analyses of extracted data were conducted iteratively and involved inductive coding and critical exploration of ideas and purposive sampling of additional papers to explore theoretical concepts in greater detail.

**Results:**

Critical analysis of 66 papers and their theoretical sources identified three key findings: (1) 9 of the 15 behavioural theories identified do not distinguish between implementation and de-implementation (5 theories were applied to only implementation or de-implementation, not both); (2) a common strategy for decreasing frequency was substituting one behaviour with another. No theoretical basis for this strategy was articulated, nor were methods proposed for selecting appropriate substitute behaviours; (3) Operant Learning Theory makes an explicit distinction between techniques for increasing and decreasing frequency.

**Discussion:**

Behavioural theories provide little insight into the distinction between implementation and de-implementation. Operant Learning Theory identified different strategies for implementation and de-implementation, but these strategies may not be acceptable in health systems. Additionally, if behaviour substitution is an approach for de-implementation, further investigation may inform methods or rationale for selecting the substitute behaviour.

**Electronic supplementary material:**

The online version of this article (10.1186/s13012-018-0826-6) contains supplementary material, which is available to authorized users.

## Background

Developing theory and evidence about interventions to support de-implementation is an important priority in implementation research. In 2014, *Implementation Science* issued an editorial arguing the need for more research to identify strategies to de-implement low-value or harmful care [1]. Since then, *Implementation Science* has published six research articles and protocols [2–7] investigating de-implementation strategies in a number of clinical contexts. Despite increasing policy interest in de-implementation, with international programmes such as the Choosing Wisely campaign [8–10] and Preventing Overdiagnosis initiative [11–13], relatively little work has been reported to understand and address systematic methods for designing de-implementation interventions. Researchers have noted de-implementation will likely involve different approaches than those used to promote people to do more of some things, but there is little evidence to support this notion [14, 15]. This raises the question of whether approaches for implementation versus de-implementation are similar or distinct. It is unknown whether or not this is the case, suggesting an investigation into whether implementation and de-implementation approaches should differ is imperative. Currently, the literature appears to lack clear guidance about what those approaches should be [16, 17].

Implementation or de-implementation as behaviour change is an important and productive thread within implementation research. A focus on reducing the frequency of overused clinical behaviours may offer a perspective that is currently lacking in the discourse on de-implementation. Behavioural theories can aid in developing a better understanding of the main effects, mediators (mechanisms), and moderators (effect modifiers) between behavioural influences and interventions in the environments (policy, system, organisation, team) [18] in which healthcare professionals work. Evidence and theory from behavioural science have informed methods for identifying factors that explain and influence behaviour and for selecting techniques to support behaviour change of healthcare professionals [19–21]. There have been major methodological and theoretical developments in the field of health psychology in designing and evaluating multi-level interventions. Advances in intervention mapping using behavioural theories have improved the design and implementation of health promotion interventions (community-level) and school-based programmes (system-level) [22, 23]. In addition, the Behaviour Change Wheel (BCW), a guide for designing interventions with its foundation in the behavioural sciences, illustrates that interventions can be delivered at any level by including policy-, system-, and individual-level components [24]. However, it is unclear to what extent theories from behavioural science propose different mechanisms for implementation and de-implementation. This study reviewed published literature to investigate whether theories of behaviour differentiate between the change processes involved in implementation and de-implementation.

The National Institute of Health defined implementation as ‘the use of strategies to introduce or change evidence-based health interventions within specific settings’ [25]. De-implementation has been broadly defined as the abandonment of medical interventions or divesting from ineffective and harmful medical practices [1]. Implementation and de-implementation interventions can be administered at any level within the healthcare system: the individual health professional, healthcare groups or teams, organisations providing health care, and the larger healthcare system [26]. The current review focused on changing what individual healthcare professionals do to improve the quality of care delivered to patients. This change can involve either doing some things more often (i.e. increasing the frequency with which a behaviour is performed, e.g. using intermittent auscultation for healthy women in labour) [27] or doing some things less often (i.e. decreasing the frequency with which a behaviour is performed, e.g. ordering X-rays for acute uncomplicated low back pain) [28].

The idea of increasing or decreasing the frequency of behaviour is relevant in other contexts. For example, facilitating people to reduce or stop harmful behaviour (e.g. stopping smoking or drug abuse) and to increase the performance of beneficial behaviours (e.g. increasing physical activity or condom use) are challenges encountered in health promotion and public health. Similarly, educators manage classroom behaviour by discouraging disruptive student actions and encouraging collaborative actions. Research in business and industry has examined strategies to reduce high-cost behaviours of employees and increase productive behaviours to improve profit margins. In yet another field, neurobiology, work has investigated different neurological pathways associated with learning and unlearning. The aims of this literature review were to explore, across diverse fields (1) whether behavioural theories differentiate between strategies for implementation and de-implementation and (2) how theory can inform processes underlying implementation and de-implementation.

## Methods

For the purpose of this study, implementation was defined as an increase in the frequency of an appropriate (evidence-based) behaviour and de-implementation as a decrease in the frequency of inappropriate (non-evidence-based) behaviour. A Critical Interpretative Synthesis (CIS) approach was used (see Table 1), whereby interpretation, critique, and insights from the literature guided the development of a theoretical rationale about a research question [29]. The CIS was conducted in three stages, described below.

**Table 1:** CIS principles as modified and applied to the current study

Purpose	• To investigate whether theory used to change behaviour differentiates conceptually between increasing and decreasing frequency of behaviour.
Process	• More closely followed traditional systematic review, but sampling, critique, and analysis were conducted concurrently.
Search strategy	• Stage 1 formal bibliographic search was foundation of the search strategy. • Research team identified key articles not identified in search. • Stage 2, theory papers were identified through those articles retrieved in Stage 1.
Sampling	• Inclusion/exclusion criteria for stage 1 were more structured and defined prior to search. • Purposive sampling of articles and other resources for stage 2 identified theory papers by the articles in the formal search, to better understand the theories and constructs.
Quality appraisal	• Not a component of this study because this was not an investigation of the effectiveness of theory use, but whether theories distinguish between increasing and decreasing behaviour.
Data analysis	• Analysis involved interrogation of the theoretical concepts that the articles reportedly used to change behaviour and the articles that reported theory development.
Findings and results	• Synthesising argument that linked theories applied to increasing and/or decreasing frequency of behaviour. • Relationship between theoretical constructs and direction of behaviour change was scrutinised. • No new constructs were generated, but new distinctions were made (between increasing and decreasing behaviour frequency).
Discussion	• Offered a theoretically sound and useful account of whether behavioural theories distinguish between increasing and decreasing frequency of behaviour. • The review was grounded in the evidence but acknowledges the ‘authorial voice’. • Some aspects of its production may not be auditable or reproducible.

### Stage 1: Identification of articles

Identification of articles followed traditional systematic review methods. Research fields that may apply behavioural theories for increasing and/or decreasing the frequency of behaviours, including psychology, health and medical sciences, education, business and marketing, law, and neurobiology, were explored.

#### Inclusion/exclusion criteria

Articles that reported the use of theory to explain changes in behaviour frequency were included if they met the following criteria:

1. Changes in behaviour were described as a change in frequency, either increasing (doing things more often) and/or decreasing (doing things less often or not at all);
2. Types of articles included were (1) reports of intervention studies (including protocols, reviews) in which theory was used to inform the development of the intervention, (2) review articles in which authors systematically reviewed the use of theory to alter behaviour frequency, (3) discussion papers that evaluated theories of behaviour change, and (4) descriptive papers in which the development or original principles of the theory was described by the original theorists;
3. The authors explicitly reported how the theory was used to inform strategies to change the frequency of behaviour under investigation.

Articles that reported or predicted behaviour of non-humans were excluded. Studies that involved behaviour change with participants with psychological pathologies such as bipolar disorder and schizophrenia, or reported pharmacological interventions, were also excluded. Articles were excluded if they reported scale development studies, measurement or programme development studies, cognitions (i.e. reported readiness or intention to change behaviour), or interventions in which behaviours were not measured (e.g. quality of life, satisfaction were measured).

#### Electronic search strategy

A list of Boolean-linked terms was constructed, covering content domains relating to (1) change in behaviour; (2) direction of change (increasing/decreasing frequency); (3) theory; (4) psychology, health and medical sciences, education, business and marketing, law, and neurobiology research areas; and (5) psychology-related terms and their synonyms (Additional file 1). We searched Academic Search Complete, PsycARTICLES, Psychology and Behavioural Sciences Collection, PsycINFO, E-Journals, CINAHL, MEDLINE, SocINDEX, GreenFILE, EconLit, Business Source Complete, Regional Business News, Teacher Reference Centre, and Criminal Justice (see Additional file 1). An initial search for peer-reviewed articles was conducted in October 2013 and a final search in March 2017. Additional articles identified by the research team based on specific knowledge of the relevant content areas were also included for screening.

#### Screening of titles and abstracts

Article titles and abstracts were screened by one researcher (AMP), and 50% were double screened and agreement calculated (Cohen’s kappa; *κ*) [30]. Where eligibility was unclear, articles were retained for full-text screening.

#### Screening of full-text papers

Full-text screening of the articles applied eligibility criteria. To be categorised as using theory, articles had to meet all three of the criteria as reported by Colquhoun and colleagues: (a) The theory had to be reported by name, referenced, and fit the definition of ‘a set of concepts and/or statements with specification of how phenomena relate to each other; (b) Theory provides an organised description of a system that accounts for what is known and explains and predicts phenomena’ (p.2) [31]; (c) The reference cited had to relate to the development of the theory and not an empirical study that cited the theory [31]. In addition, articles were required to report the complete theory, rather than a subset of constructs that would not represent the full theory. To check the reliability of full-text screening decisions made by the first researcher, random samples of articles selected from papers identified from the bibliographic search were double screened, and agreement was calculated (Cohen’s kappa; *κ*) [30].

#### Data extraction and analysis

A data extraction form was created within Microsoft Excel and piloted on four articles. Revisions to the extraction form were made to ensure the pertinent data were extracted (version 3). Data extracted included (1) the type of article (empirical or non-empirical study, review, commentary, theoretical), (2) the description of behaviour targeted for change, (3) the desired direction of behaviour change (i.e. increasing or decreasing frequency), (4) the theory reported, and (5) the cited theoretical article. Descriptive details on how the authors applied theoretical constructs or models to change behaviour frequency were also extracted.

Articles were grouped according to theory reported, and behaviours were classified according to discipline (e.g. health-related, education-related, non-specific). Similarities across the articles in which the same theory was applied were identified, and the explanatory processes proposed by the authors relating to changing behaviour frequency were compared. Strategies, interventions, or techniques that targeted theoretical constructs were identified and grouped according to direction of change.

### Stage 2: Identification of theoretical articles

To better understand the theories in articles from stage 1, the cited theoretical articles, and additional sources that may add to the interpretation and understanding of the theoretical processes proposed, were retrieved. For articles that reported the development of a theory by the original theorists, data were not formally extracted. Rather, the descriptions of processes proposed by the theorists to alter behaviour frequency were summarised and reported.

### Stage 3: Validation of theory identification

Because the search strategy likely maximised specificity rather than sensitivity, a validation process was used specifically looking for omissions. The list of theories identified in stage 2 was compared with a list of theories reported in a scoping review that purported to include theories of behaviour change from social and behavioural sciences [32]. Of particular interest were theories that were reportedly applied to both increase and decrease frequency of behaviour. Theories applied for one direction of change would not add further insight into potential differences already identified in stage 2 and were excluded.

Empirical articles identified in the scoping review [32] were evaluated for eligibility using the same criteria as applied in stage 1. The theory articles reported and cited in the included empirical articles were evaluated using the same process reported for stage 2.

### Data synthesis

Data from all included articles were synthesised to clarify the theoretical principles and how they were applied to change the frequency of behaviours. Strategies based on behavioural theories were grouped according to the direction of change (increasing or decreasing) and compared by noting the similarities and differences. Theories identified from stage 2 and the scoping review (stage 3) were grouped according to the following categories: theories applied to both increasing and decreasing frequency of behaviour, theories applied to increasing frequency of behaviours only, and theories applied to decreasing frequency of behaviour only.

## Results

### Articles retrieved through stage 1

**Fig. 1:**
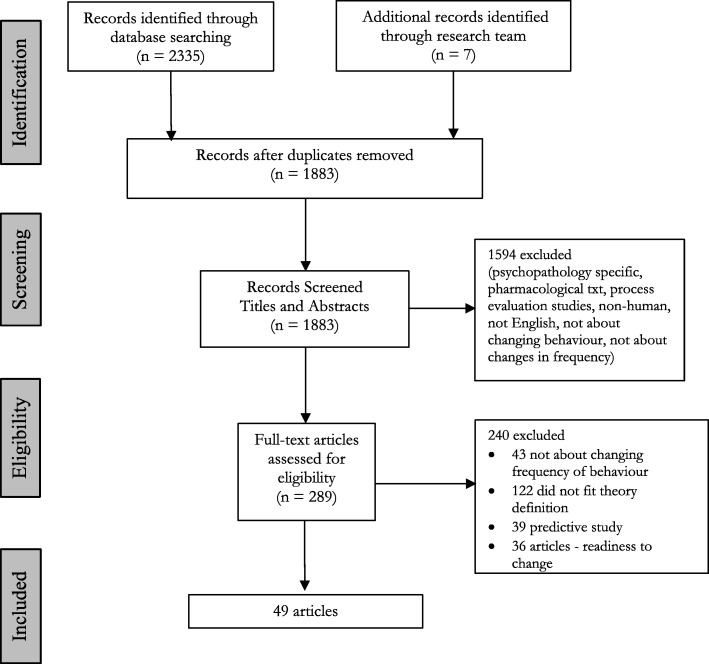
Flow diagram adapted from PRISMA for the identification of study records at stage 1 of the review

The electronic search returned 1876 articles after the removal of duplicates (Fig. 1) with 7 articles identified through other sources (*n* = 1883). Screening of titles and abstracts resulted in the exclusion of 1594 articles, leaving 289 articles for full-text screening. Full-text screening resulted in the exclusion of 240 articles. Fifty-four articles were double screened to check reliability of inclusion criteria. Agreement between coders of screening titles and abstracts and of full-text screening was *κ* = 0.78 and *κ* = 0.80, respectively, indicating substantial agreement [33].

### Data extraction from stage 1 articles

Summaries of the data extracted from the 49 included articles are reported in Table 2. Briefly, 32 articles were empirical studies, 6 were protocols, 4 were commentary/discussion papers, 4 were review studies, and 3 were articles about theory development. The majority of studies applied behavioural theories to health and public health research (*n* = 35), whilst 6 studies reported the application of theories to psychology research. Eight studies applied theories to education, law, health profession, and neurobiology research (2 in each research field). When describing the change in behaviour frequency, 24 articles described increasing the frequency of behaviour, 8 described decreasing frequency, whilst 17 articles reported multiple behaviours and targeted both increasing and decreasing frequencies.

**Table 2:** Characteristics of articles included in CIS review from stage 1

Characteristics of articles	Number of articles (*n* = 49)
Type of article	
Empirical	32
Protocol	6
Commentary/discussion	4
Review	4
Theory development	3
Description of behaviours	
General	8
Specific behaviours	41
Research area theory was applied	
Education	2
Health and public health	35
Law	2
Health professional	2
Neurobiology	2
Psychology	6
Direction of behaviour change	
Increasing frequency	24
Decreasing frequency	8
Both increasing and decreasing frequencies	17
Theories reported*	
Control Theory	1
Deterrent Theory	2
Disconnected Value Model	5
Goal-Setting Theory	1
Health Action Process Approach	2
Health Belief Model	1
Implementation Intention	4
Operant Learning Theory	2
Protection Motivation Theory	1
Self-Affirmation Theory	1
Self-Determination Theory	5
Social Cognitive Theory	23
Temporal Self-Regulation Theory	1
Theory of Planned Behaviour	4
Theory of Reasoned Action	2

*Eight articles reported the application of more than one theory; therefore, the sum of the theories reported column is greater than 49

Fifteen behavioural theories were reportedly applied or proposed to increase and/or decrease the frequency of behaviour (Control Theory [34], Deterrent Theory [35], Disconnected Value Model [36], Goal-Setting Theory [37], Health Action Process Approach [38], Health Belief Model [39], Implementation Intention [40], Operant Learning Theory [41], Protection Motivation Theory [42], Self-Affirmation Theory [43], Self-Determination Theory [44], Social Cognitive Theory [45], Temporal Self-Regulation Theory [46], Theory of Planned Behaviour [47], and Theory of Reasoned Action [48]).

### Articles retrieved at stage 2

The theoretical articles (*n* = 15) cited in the 49 empirical articles were retrieved, as well as additional resources to further aid in understanding the theories, constructs, and their application to changing the frequency of behaviour (e.g. psychology resource books, cited articles in the theoretical articles).

### Stage 3: Validation of theory identification

**Fig. 2:**
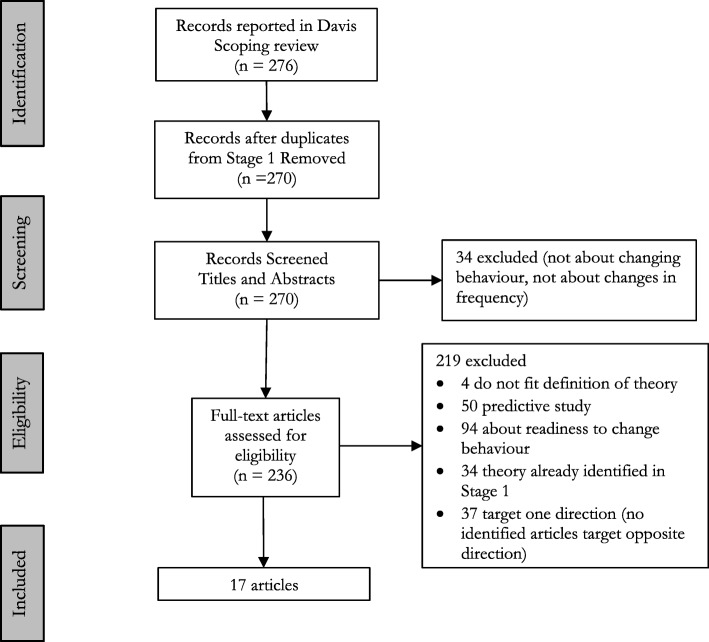
Flow diagram adapted from PRISMA for the identification of articles from scoping review [32]

Of the 276 articles reported in the scoping review [32], 270 were included for screening of title and abstract (duplicates from stage 1 removed). Screening of titles and abstracts resulted in exclusion of 33 articles. Full-text screening of the remaining 237 articles resulted in exclusion of 209 articles (Fig. 2). Twenty-two theories not identified in stage 1 were identified in 33 articles but changed behaviour in only 1 direction (i.e. 30 articles targeted increasing behaviour frequency; 3 targeted decreasing behaviour frequency). No articles in the scoping review applied these theories to change behaviour frequency in both directions.

One theory was added as a result of this validation procedure. The information-motivation-behavioural (IMB) skills model [49] was applied to increasing and decreasing frequency and reported in 17 articles. All 17 articles reported the application of IMB skills model to health behaviour research. Detailed descriptions of the included studies from stage 1 to stage 3 are presented in Additional file 2.

### Data synthesis

The identified theories were grouped into three categories: (1) theories applied to both *increasing and decreasing behaviour*, (2) theories applied to increasing behaviour only, and (3) theories applied to decreasing behaviour only (Table 3). Descriptions of the theories are presented in Additional file 3.

**Table 3:** Summary of theories reported in articles by direction of change in behaviour frequency

Theories/models applied to increase or decrease frequency of behaviour	Target: increasing frequency	Target: decreasing frequency	Different directions theorised differently?
Operant Learning Theory	Yes	Yes	Yes
Implementation Intention	Yes	Yes	No*
Social Cognitive Theory	Yes	Yes	No*
Disconnected Value Model	Yes	Yes	No*
Self-Affirmation Theory	Yes	Yes	No*
Self-Determination Theory	Yes	Yes	No*
Theory of Planned Behaviour	Yes	Yes	No*
Theory of Reasoned Action	Yes	Yes	No*
Temporal Self-Regulation Theory	Yes	Yes	No*
Information-Motivation-Behaviour Skills Model^a^	Yes	Yes	No*
Deterrent Theory	No	Yes	N/A
Control Theory	Yes	No	N/A
Goal-Setting Theory	Yes	No	N/A
Health Action Process Approach	Yes	No	N/A
Health Belief Model	Yes	No	N/A
Protection Motivation Theory	Yes	No	N/A

^a^Models/theories identified from scoping review

*Proposed decreasing an undesired behaviour by attempting to increase a substitute behaviour

#### Theories applied to both increasing and decreasing frequency of behaviours

Ten theories were grouped into two categories: theories that propose different approaches to increasing and decreasing behaviour, and theories that do not.

##### Theories that propose different approaches for increasing and decreasing behaviour

Only one theory proposed different strategies for increasing and decreasing behaviours, Operant Learning Theory (OLT). Experimental studies based on Operant Learning Theory (OLT) supported the hypothesis that different approaches are effective for increasing [50, 51] and decreasing [52, 53] the frequency of behaviours. For example, in one study, OLT was used to explore the neurobiological connections of reinforcement (i.e. administering a positive stimulus if and only if the behaviour was performed) and punishment (i.e. aversive stimulus delivered if and only if the behaviour was performed). Individuals were involved in an instrumental learning task (i.e. simple learning task of pressing (or not pressing) a button in the presence of an image on a computer screen) [52]. Participants were rewarded for pressing a button when a specific image was presented on a computer screen (to increase this behaviour) or punished for pressing a button when a different image was presented on a computer screen (to decrease this behaviour) [52]. Studies in the neurobiology of behaviour change illustrated that different neurological pathways may be responsible for different directions of change in behaviour frequency. Specifically, the neurotransmitter dopamine is involved in the activation of behaviour whilst serotonin appears to be more closely associated with behavioural inhibition [51, 52, 54, 55].

Despite OLT proposing different strategies for implementation and de-implementation, some authors used reinforcement strategies to decrease undesired behaviours by reinforcing a substitute behaviour that was incompatible with the problematic behaviour. For example, Epstein and colleagues [53] recommended parents give praise (positive reinforcements) to children whenever they ate fruit and vegetables or exercised, regardless of whether the target behaviour was to ‘increase fruit and vegetable intake’ (implementing behaviour) or to ‘decrease fat intake’ (de-implementing behaviour).

##### Theories that do not propose different approaches for increasing and decreasing behaviour

These theories were Disconnected Values Model (DVM) [56], IMB skills model [57], Implementation Intention (II) [40], Self-Affirmation Theory [43], Self-Determination Theory [58], Social Cognitive Theory (SCT) [45], Theory of Planned Behaviour [47], Theory of Reasoned Action [48], and Temporal Self-Regulation Theory [46].

To increase the frequency of behaviour, Anshel et al. [36] through DMV proposed that when positive habits align with an individual’s values and desires, the positive habit/behaviour will continue. IMB skills model [57] was reportedly used to increase medication adherence, physical activity, and condom use [59–61]. IMB skills model targets improving individuals’ knowledge base, motivation, and skills about a behaviour in order to increase the likelihood of performing the behaviour [62–64]. Schweiger Gallo and Gollwitzer [65] and Orbell et al. [66] illustrated that clearly defined plans about when and in what context a behaviour will be performed (II) increase frequency of behaviour. Self-Affirmation Theory was used to increase healthy behaviour such as exercise and healthy eating by proposing behaviours will increase if they align with an individual’s self-worth and values [67]. Self-Determination Theory proposes a process whereby behaviour occurs when focusing on intrinsic motivation as well as the individual’s need to feel competent, in control of one’s own life, and to be connected to others. By focusing on these needs as they relate to health behaviour, several authors proposed that physical activity [68, 69] and oral care [70] will increase. The authors who used SCT targeted increasing individuals’ self-efficacy and specifying goals about the target behaviour. Ranby et al. [71] applied SCT with the Health Belief Model through discussions about the health threats of being overweight in a highly stressful job. They noted the importance of setting goals and monitoring to improve the individual’s self-efficacy to achieve the desired behaviour of increasing exercise. Similarly, other researchers improved students’ self-efficacy through goal setting about eating a target number of fruits per week in an attempt to increase fruit consumption [72, 73]. Both the Theory of Planned Behaviour and Theory of Reasoned Action were applied to increase individuals’ intentions about exercise and healthy eating [74, 75] as well as condom use [76]. Lastly, Borland discussed the application of Temporal Self-Regulation Theory to increase and maintain behaviours and that by focusing on long-term benefits rather than immediate outcomes individuals are more likely to perform adaptive behaviours and continue the behaviour [77].

To decrease frequency of behaviours, authors who applied theories that did not theorise decreasing differently from increasing, proposed replacing the undesired behaviour with a new desired behaviour. The principles of the identified theories were then applied to increase the frequency of the substitute behaviour. For example, Anshel suggested replacing negative habits with positive routines and emphasising the values associated with positive routines [56]. Using Self-Determination Theory, Weber-Gasparoni et al. [70] recommended parents replace sugary food and drinks with healthier ones to improve oral care in children. Using II and SCT, Albright and colleagues and Armitage suggested participants develop positive plans or implementation intentions rather than negative ones (e.g. ‘I will eat more fruits this week’ versus ‘I will stop eating meat this week’) to decrease fat intake [73, 78]. Avants and colleagues [79] replaced ‘harmful behaviours’ (unprotected sex) with a safer behaviour (increase condom use) and used the IMB skills model to increase condom use. Similar recommendations of a substitute behaviour were provided by authors who applied Self-Affirmation Theory to reduce caffeine and alcohol consumption [67], Theory of Planned Behaviour to reduce antibiotic prescribing [80, 81], Theory of Reasoned Action to reduce fat consumption [82], and Temporal Self-Regulation Theory in smoking cessation [77].

#### Theories only applied to increase behaviours

Control Theory [34], Goal-Setting Theory [37], Health Action Process Approach (HAPA) [38, 83], Health Belief Model (HBM) [39], and Protection Motivation Theory (PMT) [42] generally focused on factors that improved individuals’ performance if behaviour of motivation is high. For example, Gray et al. [84] proposed that elements of Control Theory such as self-monitoring, planning, goal setting, and review, as well as feedback on behaviour, could increase the participants’ physical activity. Le [85] using HBM hypothesised that perceived weight gain would act as a ‘cue to action’, thereby increasing the likelihood of physical activity. Using the HAPA model, Fleig et al. [86] reported that individuals who generated plans to increase physical activity or eating fruit and vegetables were more likely to perform the behaviour described in those plans. Similarly, Ivers et al. [87], using Goal-Setting Theory and planning reported that physicians would likely act to improve quality of patient care if clearly define goals and actions plans were in place. A systematic review conducted by Bish and colleagues [88], examining interventions to increase H1N1 vaccination rates, applied PMT to evaluate how the perceived severity and personal risk of H1N1 pandemic may increase subsequent uptake of the vaccine.

#### Theories only applied to decrease behaviours

Deterrent Theory (DT) applies the expectation of punishment to discourage youth offenders from reoffending (e.g. underage alcohol consumption, drug use) [35, 89, 90]. Maxwell proposed that the sole purpose of punishment issued by criminal law-enforcing bodies is to deter future crimes, a principle of the judicial system [89, 91].

## Discussion

This study aimed to investigate whether behavioural theories propose different approaches to increasing (implementation) or decreasing (de-implementation) behaviours. Three main findings emerged from the synthesis of the included articles. First, most behavioural theories do not differentiate between increasing and decreasing frequency of behaviour. It is possible that this position is defensible. If this is the case then future investigation should involve determining if certain theories are better for implementation or de-implementation. Behavioural theories provide different ways of explaining behaviour, focusing on different factors, determinants, or constructs. Some theories are better at explaining how behaviours are formed or implemented than others, depending on the most influential determinant, i.e. learning theories explain how individuals learn new behaviours, motivational theories such as SCT and TPB, identify the factors that may determine motivation, and action theories such as HAPA and Control Theory, help in following through from intention to behaviour, can facilitate habit formation. It may be necessary to investigate whether propositions hold for de-implementing behaviours. Designing interventions to target behaviour change may be enhanced by identifying whether there is a difference in predictive validity of theories that do, compared with those that do not, distinguish between processes for implementation and de-implementation.

Second, many studies using theories that do not differentiate between implementing and de-implementing behaviours applied the strategy of selecting a substitute behaviour to reduce an undesired behaviour. Authors did apply theory to increase the frequency of the substitute behaviour. No theoretical rationale for this substitution strategy was reported, and no methods for selecting appropriate substitute behaviours were proposed. This approach first requires the selection of an appropriate substitute behaviour. Behaviour substitution is not a new concept for reducing behavioural frequency and is an established behaviour change technique [19]. When used with reinforcements to increase the frequency of the substitute behaviour this strategy is termed *differential reinforcement of an incompatible behaviour* (DRI) [92, 93], a behaviour modification strategy that directly applies the principles of OLT. The goal of DRI is to reinforce only those responses that are desirable [94] with a view to reducing the performance of the undesired behaviour. The articles in this review that reported a behaviour substitution strategy did not mention DRI or the theoretical rationale for using this technique. Rather, selection of the substitute behaviour appeared to be based on intuitive principles and theory was used to target the substitute behaviour. Further, no methods were proposed to guide the selection of an appropriate substitute behaviour. In clinical practice, there may be no obvious substitute behaviour. For example, evidence-based clinical recommendations suggest that healthcare providers stop doing something (i.e. ‘ordering chest X-rays for healthy patients having elective surgeries’) without suggesting substitute behaviours to increase. Applications of this strategy may therefore require additional investigation among different healthcare professional (HCP) groups and behaviours to determine its potential generalizability as a de-implementation strategy.

Third, OLT was the only theory to propose different approaches for increasing versus decreasing frequency of behaviour. The basic principles of OLT are that a behaviour will occur more frequently if it is followed by reinforcement [50]. Conversely, behaviour will occur less frequently if it is followed by punishment. However, there are several challenges to applying the principles of OLT to changing healthcare behaviours, which involves applying OLT principles initially tested with animals in laboratory settings to human participants in complex, real world situations.

There are at least four reasons why applying OLT in these settings may be problematic. First, in complex situations, it is highly likely that other behaviours will be performed between the performance of the target behaviour and delivery of the reinforcement or punishment, so the link between the two is often obscured. Reinforcements and punishments are most effective when they immediately follow the behaviour. However, the results (either positive or negative) of most HCPs’ behaviour may occur days, weeks, or months after the behaviour has been performed. Second, applying OLT to changing HCPs’ behaviours is likely to be most effective if all contingencies of behaviour can be understood (including its ‘antecedents’ and the specific environmental conditions in which a behaviour will be rewarded); only then is it possible to predict and control behaviour [41].

A third challenge with applying OLT is that there is a poor evidence base to select the dose, or potency, of stimulus required to have an effect. It is unclear whether there is a linear relationship between potency of the stimulus and behaviour change. The strength of the stimulus needed to reinforce is often less intense than the strength of the stimulus needed to punish to elicit the same level of effect [41, 95, 96]. For example, disciplinary actions or sanctioning of an HCP’s medical practice is utilised by these agencies to reduce or stop HCP behaviour can be seen as a form of punishment. De-accreditation is used in extreme cases of professional misconduct but not for day-to-day practice errors. Fourth, the ethics and equity of applying conditional rewards and conditional punishments to healthcare professionals are currently unclear. The lack of utilisation of punishments in HCP behaviour change may likely be because it goes against the concept of clinical autonomy and self-regulation within professional bodies. If we think of ‘punishment’ as having to have a conversation with the clinical manager, or being asked to re-submit a test ordering form, the ethical problems start to become less extreme. Further investigation is needed into these uncertainties in order to determine the usefulness of OLT as a possible approach for de-implementation.

### Strengths and limitations

This review set out to systematically explore whether behavioural theories differentiate between mechanisms involved in increasing and decreasing frequencies of behaviour change. An integral aspect of the CIS method involved critical reflection about the articles included in the review and exploration of themes and ideas through purposive sampling of relevant papers. The strategy, whereby a structured systematic electronic search of literature was supplemented with the inductive, iterative, and purposive sampling of articles, allowed for transparency and rigour whilst maximising insight. However, the very nature of CIS implies that there is a level of subjectivity in the interpretation that is not necessarily evident in other types of reviews. Researchers with different expertise than those of the current research team, attempting to replicate these findings, may have different interpretations arising from their own knowledge base. Nonetheless, we would argue that this review has identified important findings that may inform this field.

The focus of the search strategy was to identify those papers that explicitly reported both behaviour change and the use of theory to explain the behaviour change. Because of this narrow focus, many papers were not included. Other researchers using the CIS approach may decide to be more inclusive in their selection criteria, including studies like those in scoping reviews that were excluded from this review [32]. Additional theories may have been identified in the excluded papers. For example, papers were excluded from this study if intention to change was evaluated, rather than actual behaviour. In addition, despite claiming that theory was applied to their study design, few authors reported the explicit use of theory. For example, when describing strategies for changing behaviour frequency, several authors did not clearly specify their theoretical rationale (e.g. [97–99]). There was often no direct link between the theory proposed by the authors and the techniques reported for changing behaviour (e.g. [100–102]). Despite the absence of these articles in the review, a number of theories were presented that may inform different processes (OLT) and techniques (behaviour substitution) to support implementation and de-implementation.

The inclusion of articles that described theories, or their development, ensured that less frequently reported empirically tested theories were not excluded simply because they had not been as rigorously tested as other theories. The focus of the review was a conceptual synthesis of theories rather than empirical testing of theories. There was no formal evaluation of the quality of the empirical evidence reported in the included papers, nor was the search exhaustive for all possible evidence of a theoretical basis for designing interventions differently based on direction of change. However, the objective was to reach a level of saturation, and the results of the study suggest that this was achieved because of the limited number of studies added from the scoping review in stage 3 of this review.

Unless an implementation intervention that is delivered at system-level or organisational-level actually changes the care that a patient receives from healthcare teams and individual healthcare professionals, it fails to enhance care quality and therefore fails to improve health outcomes. A strength of the review is the focus on behaviours of healthcare professionals and teams, no matter where in a healthcare system an intervention is delivered. Wang et al. proposed four different types of de-implementation related to organisational effort (partial reversal, complete reversal, related replacement, unrelated replacement) [103]. Behaviour theories may help inform any of these four types, since the underlying foundation of all four is removing ineffective practice and performing the associated behaviour less often. The first two types focus on reducing the frequency of behaviour from either (i) often to not at all for a sub group of patients (partial reversal—removing ineffective practice) or (ii) often to not at all for the whole patient population (complete reversal—removing ineffective practice). The latter two types (related replacement and unrelated replacement) propose a potential strategy (behaviour substitution) for de-implementation. As we have highlighted in the current review, behaviour substitution is a behaviour change technique [22] that has been used to decrease an undesired behaviour [56, 67, 70, 73, 77–81]. However, methods for identifying and targeting a substitute behaviour are currently underdeveloped and require further investigation. Behavioural theories can be applied to enhance the uptake of the selected substitute behaviour.

One area of psychology that is absent from this review is the field of cognitive psychology. Cognitive psychology research has reported that decisions to act followed by a negative outcome produce more regret (action regret) than decisions to refrain from acting followed by a negative outcome (inaction regret) in the short term [104]. However, inactions give rise to more intense regret over time [104–106]. This suggests that there are temporal asymmetries in the emotional consequences of negative outcomes that were associated with the direction of behaviour change. Directly after an outcome, actions are noticeable and more likely to be internally acknowledged than are inactions [107]. However, these perceptions of responsibility may change. When people think back upon actions which resulted in bad outcomes, they may think ‘At least I tried; it was all I could do’, and possibly reduce the sense of responsibility from the bad outcome [104, 106]. This may be particularly important for changing the behaviour of healthcare professionals. The potential negative outcomes from de-implementation interventions (inaction; e.g. not to prescribe unnecessary drugs) may be associated with greater regret than the potential negative outcomes from implementation (action; e.g. to order bone mineral density scans for patients over 50 years of age with a fracture). Negative outcomes in healthcare can be life threatening to the patient. The perception that healthcare professionals ‘did nothing’ (inaction) may be associated with greater regret if the consequences are negative than if it is perceived that health professionals did ‘everything they could’ (action). Further work with the application of cognitive psychology to implementation and de-implementation interventions is required.

## Conclusion

This review identified a range of behaviour change literature that purports to invoke theory. The majority of theories do not propose different approaches for implementation and de-implementation. Furthermore, although the strategy of increasing a substitute behaviour to replace an undesired behaviour was often used, no study reported a rationale for this. Currently, there do not appear to be systematic methods for selecting appropriate substitute behaviours. Exploration of the selection and use of substitute behaviours needs further investigation and conceptualisation. We did note that Operant Learning Theory (OLT) proposes different strategies for increasing or decreasing behaviour frequency and some initiatives already utilise aspect of OLT (e.g. payment for services, such as the NHS Quality and Outcomes Framework, and extreme cases of disciplinary actions or sanctioning of HCP practice). However, the effects, including unintended effects, of OLT are not well understood. In addition, punishment except in those extreme cases is not systematically used, and this may be a missed opportunity. In view of the imperative to increase efficiency in health systems by reducing low-value care, further research should work towards more robust methods for designing de-implementation interventions.

## Additional files

Additional file 1: Search terms used and databases searched for stage 1. (DOCX 43 kb)

Additional file 2: Articles included in CIS review that reported the application of theory to change frequency of behaviour. (DOCX 81 kb)

Additional file 3: Theory descriptions reported to theorise strategies for changing frequency of behaviour. (DOCX 36 kb)

## Abbreviations

CIS: Critical Interpretive Synthesis
DMV: Disconnected values model
DRI: Differential reinforcement of an incompatible behaviour
DT: Deterrent Theory
HAPA: Health Action Process Approach
HBM: Health belief model
HCP: Healthcare professional
II: Implementation intention
IMB: Information-motivation-behavioural skills
OLT: Operant Learning Theory
SCT: Social Cognitive Theory
