# Supplementary material for: Changing behaviour ‘more or less’—do theories of behaviour inform strategies for implementation and de-implementation? A critical interpretive synthesis
Source: Implement Sci. 2018 Oct 29;13:134. doi: 10.1186/s13012-018-0826-6 (PMC6206907; doi:10.1186/s13012-018-0826-6)
Supplement: Supplementary file 1 — Search terms used and databases searched for stage 1. (DOCX 43 kb) [file 13012_2018_826_MOESM1_ESM.docx]

Additional file 1: Search terms used and databases searched for Stage 1

Note: terms listed do not include all MESH terms and synonyms used

Theory or theory-based or model

AND

Change N3 practice" or action or management or "behavio#r nr1 change"

AND

Increas* OR Decreas* OR Improv* OR Promot* OR Enhanc* OR Optimi#* OR Diminish* OR Reduc* OR Activate OR Inhibit OR "Stamp* nr1 in" OR "Stamp* nr1 out" OR Learning OR Unlearning OR Reward OR Punishment OR Extinction OR "Encouraging nr1 good nr1 behavio#r" OR "Discouraging nr1 bad nr1 behavio#r" OR Prompt OR Remind

AND

"Sympathetic nr1 nervous n1 system" OR "Parasympathetic nr1 nervous nr1 system" OR "Excitatory nr1 response" OR "Inhibitory nr1 response" OR "Neurobiology nr3 extinction" OR Homeostasis OR Self-regulation OR "Cognitive nr1 associations" OR Cessation OR Dietary OR Exercise OR Prescrib* OR Adherence OR Intervention OR "Physical nr1 Activity" OR "Guideline nr1 Adherence" OR "Guideline nr1 compliance" OR "Quality nr1 improvement" OR "Classroom nr1 management" OR "Marketing nr1 behavio#r" OR "Consumer nr1 behavio#r" OR "Choice nr1 concept" OR “Enterprise” OR “Behavio#r nr1 modification” OR "Conflicting nr1 behavio#r" OR "Reinforced nr1 behavio#r" OR Habit OR "Habit nr1 reversal" OR "Operant nr1 learning" OR "Classical nr1 conditioning" OR "Action nr1 regret" OR "Inaction nr1 regret" OR Commission OR Omission OR Reward OR Punishment OR "Dual nr1 Process nr1 model*" OR "Reflective nr1 learning" OR "Associative nr1 learning" OR "High nr1 cognitive nr1 load" OR "low nr1 cognitive nr1 load" OR "Regulatory nr1 Focus" OR "Positive nr1 feedback" OR "Negative nr1 feedback"

| Fields of Research  Databases | Psychology | Education | Health Policy, Promotion & Behaviour | Business & Marketing | Neurobiology |
| --- | --- | --- | --- | --- | --- |
| Academic Search Complete | ✔ | ✔ | ✔ | ✔ | ✔ |
| PsycARTICLES | ✔ | ✔ | ✔ | ✔ | ✔ |
| Psychology and Behavioural Sciences Collection | ✔ | ✔ | ✔ | ✔ | ✔ |
| PsycINFO | ✔ | ✔ | ✔ | ✔ | ✔ |
| E- Journals | ✔ | ✔ | ✔ | ✔ | ✔ |
| CINAHL | ✔ |  | ✔ |  |  |
| MEDline | ✔ |  | ✔ |  | ✔ |
| SocINDEX |  | ✔ | ✔ | ✔ |  |
| GreenFILE |  |  |  | ✔ |  |
| EconLit |  |  |  | ✔ |  |
| Business Source Complete |  |  |  | ✔ |  |
| Regional Business News |  |  |  | ✔ |  |
| Teacher Reference Centre |  | ✔ |  |  |  |
| Criminal Justice | ✔ |  |  |  |  |
| **NOTE: Check mark indicates the database related to the research discipline** | | | | | |
