# Supplementary material for: Changing behaviour ‘more or less’—do theories of behaviour inform strategies for implementation and de-implementation? A critical interpretive synthesis
Source: Implement Sci. 2018 Oct 29;13:134. doi: 10.1186/s13012-018-0826-6 (PMC6206907; doi:10.1186/s13012-018-0826-6)
Supplement: Supplementary file 2 — Articles included in CIS review that reported the application of theory to change frequency of behaviour. (DOCX 81 kb) [file 13012_2018_826_MOESM2_ESM.docx]

**Additional file 2****: Articles included in CIS review that reported the application of theory to change frequency of behaviour**

| Author and year of publication | Type of article | Description of Behaviour under investigation | Direction of frequency change | Theory/ies | Theory Author and Year |
| --- | --- | --- | --- | --- | --- |
| (Anshel et al., 2010a) | Empirical | Non-descript negative actions or habits (examples not provided) | Decrease | Disconnected Values Model | (Anshel, 2008) |
| (Anshel, 2010) | Empirical | Unhealthy habits*, physical activity  *Unhealthy habits were not evaluated b/c directing unclear with respect to the habits were actions or in actions. | Increase |  |  |
| (Anshel, 2013) | Empirical | Physical activity | Increase |  |  |
| (Anshel et al., 2010b) | Empirical | Physical activity | Increase |  |  |
| (Anshel, 2008) | Development of theory | Negative actions & positive habits | Increase and decrease |  |  |
| (Fleig et al., 2011) | Empirical | Physical activity, fruit and vegetable consumption | Increase | Health Action Process Approach | (Schwarzer, 2008) |
| (Schwarzer, 2008) | Development of theory | Health-enhancing behaviours (uses examples such as physical activity, breast self-exam, seat-belt use, healthy eating, and dental flossing – **all increasing behaviours) *included health-compromising behaviour but did not provide examples** | Increase |  |  |
| (Armitage, 2004) | Empirical | Reducing fat intake | Decrease | Implementation Intentions | (Gollwitzer, 1993) |
| (Schweiger Gallo & Gollwitzer, 2007) | Review | Multiple behaviour | Increase and decrease |  |  |
| Orbell et al., 1997 (Orbell et al., 1997) | Empirical | Breast self exam | Increase |  |  |
| (Avants et al., 2004) | Empirical | High risk behaviour (Unprotected sex; using dirty drug paraphernalia) | Decrease |  |  |
| (Amico et al., 2005) | Empirical | Adhering to AZT drug | Increase | Information-Motivation-Behavioural (IMB) Skills Model | (Fisher & Fisher, 1992b) |
| (Anderson et al., 2006) | Empirical | Using condom |  |  |  |
| (Cornman et al., 2007) | Empirical | Condom use |  |  |  |
| (Fisher et al., 1994) | Empirical | Condom use |  |  |  |
| (Fisher et al., 1996) | Empirical | Condom use |  |  |  |
| (Fisher et al., 2002) | Empirical | Condom use |  |  |  |
| (Fisher & Fisher, 1992a) | Development of theory | General behaviour (no examples provided) |  |  |  |
| (Jaworski & Carey, 2001) | Empirical | Condom use |  |  |  |
| (Kalichman et al., 2002) | Empirical | Electronic searches for HIV/AIDS Health information |  |  |  |
| (Kalichman et al., 2006) | Empirical | Condom use/drug adherence |  |  |  |
| (Kiene & Barta, 2006) | Empirical | Condom use/drug adherence |  |  |  |
| (Osborn et al., 2010) | Empirical | Physical activity |  |  |  |
| (Rye et al., 2008) | Empirical | Condom use |  |  |  |
| (Simon et al., 2010) | Empirical | Drug adherence |  |  |  |
| (Yang et al., 2011) | Empirical | Condom use |  |  |  |
| (Zarani et al., 2010) | Empirical | Multiple health behaviours |  |  |  |
| (Stone, 1953) | Empirical | Decrease behaviour in serial activities (experiments) | Decrease | Operant Learning Theory | (Skinner, 1963); (Thorndike, 1927);  (Skinner, 1976) |
| (Crossley et al., 2012) | Empirical | Procedural skills | Decrease |  |  |
| (Strohacker et al., 2014) | Review | Exercise behaviour (described as attendance at exercise sessions, physical activity counts, steps per day, meeting weekly gym goal visits) | Increase |  |  |
| (Guitart-Masip et al., 2012) | Empirical | Simple learning task | Increase and decrease |  |  |
| (Deforche et al., 2011) | Commentary/ Discussion | Physical activity | Increase | Self-Determination Theory | (Deci & Ryan, 1985);  (Ryan & Deci, 2000) |
| (Gillison et al., 2014) | Empirical (protocol) | Physical activity and food intake (food intake was not evaluated because direction unclear with respect to eating more fruit and vegetable and eating less high fat) | Increase |  |  |
| (Lubans et al., 2012) | Empirical (protocol) | Physical activity | Increase |  |  |
| (Weber-Gasparoni et al., 2013) | Empirical | Oral care (daily tooth brushing, bedtime tooth brushing, use of fluoridated toothpaste, pea size toothpaste used, drinking sugary drinks, child eating more than 2 cariogenic snacks/day, checking for early signs or caries) | Increase and decrease |  |  |
| (Strecher et al., 1995) | Commentary/ Discussion | Fat intake and smoking | Decrease | Social Cognitive Theory | (Bandura, 1976; Bandura, 1977; Bandura, 1986, 1998, 2004) |
| (Bandura, 1976) | Development of theory | Non-descript behaviour (examples not provided) | Increase |  |  |
| (Barling, 1980) | Empirical | Answering questions on verbal and arithmetic tests | Increase |  |  |
| (Craciun & Baban, 2008) | Empirical | Fruit intake | Increase |  |  |
| (Fox & Bailenson, 2009) | Empirical | Exercise activity | Increase |  |  |
| (Coday et al., 2002) | Empirical | Physical activity | Increase |  |  |
| (Sorensen et al., 1998) | Empirical (protocol) | Fruit and Vegetable consumption | Increase |  |  |
| (Moore et al., 2006) | Empirical | Exercise | Increase |  |  |
| (Pérez-Rodrigo et al., 2005) | Empirical | Fruit and vegetable intake | Increase |  |  |
| (Smith et al., 2010) | Empirical | Physical activity and food intake*  * Food intake was not evaluated b/c direction unclear with respect to eating more fruit and eating less high fat. | Increase |  |  |
| (Albright et al., 1997) | Empirical | Consuming fat and eating fruit and vegetables | Increase and decrease |  |  |
| (Anderson-Bill et al., 2011) | Empirical | Daily intake of calories, daily servings of fruit and vegetables, daily steps | Increase and decrease |  |  |
| (Crockett et al., 1988) | Empirical | Healthy eating, food intake - fruit, vegetables and fatty foods | Increase and decrease |  |  |
| (Howard-Pitney et al., 1997) | Empirical | Fat consumption and fruit and vegetable consumption | Increase and decrease |  |  |
| (Lubans et al., 2010) | Empirical | Physical activity, frequency of food item consumption | Increase and decrease |  |  |
| (Macaulay et al., 1997) | Empirical | Healthy activity (physical activity frequency) and food intake (sugar, fat, and fruit and vegetable consumptions) | Increase and decrease |  |  |
| (Paradis et al., 2005) | Empirical | Healthy activity (physical activity frequency) and food intake (sugar, fat, and fruit and vegetable consumptions) | Increase and decrease |  |  |
| (Barratt et al., 2005) | Empirical | Obtaining cannabis | Decrease | Deterrence Theory | (Schneider & Ervin, 1990) |
| (Maxwell & Gray, 2000) | Empirical | Commit crimes | Decrease |  |  |
| (Griffin et al., 2011) | Empirical (protocol) | Physical activity, dietary intake (fruit and vegetable intake), medication adherence, smoking cessation | Increase and decrease | Theory of Planned Behaviour; Other theories were reported but did not fit definition of use (used one construct in theory) | (Ajzen, 1991) |
| (Griffin et al., 2014) | Empirical |  |  |  |  |
| (Hrisos et al., 2008a) | Empirical (protocol) | Antibiotic prescribing | Decrease | Theory of Planned Behaviour, Social Cognitive Theory | (Ajzen, 1991); (Bandura, 1998) |
| (Hrisos et al., 2008b) | Empirical |  |  |  |  |
| (Harris, 2011) | Commentary/ Discussion | Unhealthy behaviours (smoking, caffeine consumption, alcohol consumption) & Healthy behaviour exercise, fruit and vegetable consumption) | Increase and decrease | Self-Affirmation Theory | (Steele, 1988) |
| (Borland, 2010) | Commentary/ Discussion | Non-descript behaviour in general | Increase and decrease | Temporal Self-regulation Theory | (Hall & Fong, 2007) |
| (Burke et al., 2002) | Empirical | Exercise, fruit and vegetable consumption, fat consumption | Increase and decrease | Social Cognitive Theory; Theory of Reasoned Action | (Bandura, 1986); (Fishbein & Ajzen, 1975) |
| (Fleig et al., 2011) | Empirical | Physical activity, fruit and vegetable consumption | Increase | Health Action Process Approach | (Schwarzer et al., 2008) |
| (Schwarzer, 2008) | Development of theory | Health-enhancing behaviours (uses examples such as physical activity, breast self-exam, seat-belt use, healthy eating, and dental flossing – **all increasing behaviours) Health compromising behaviour were mentioned but not discussed** | Increase |  |  |
| (Gray et al., 2013) | Empirical | Physical activity and healthy eating*  *Healthy eating was not evaluated b/c direction unclear with respect to eating more fruit and eating less high fat. | Increase | Control Theory | (Carver & Scheier, 1990) |
| (Ivers et al., 2010) | Empirical (protocol) | Test ordering and prescription rates | Increase | Goal Setting Theory; Implementation Intentions | (Locke & Latham, 1994); (Gollwitzer, 1999) |
| (Pulley et al., 1996) | Empirical | Condom use, use of bleach to disinfect needles the reduce risk of HIV infection | Increase | Social Learning Theory, Health Belief Model; Theory of Reasoned Action | (Bandura, 1986); (Rosenstock, 1974); (Fishbein & Ajzen, 1975) |
| (Ranby et al., 2011) | Empirical | Exercise, fruit and vegetable consumption | Increase | Social Cognitive Theory; Health Belief Model | (Bandura, 1986); (Rosenstock, 1974) |
| (Godin & Shephard, 1990) | Review | Exercise | Increase | Protective Motivation Theory  (SCT, HBM, TRA, TIB, TPB were identified in review but was predictive studies) | (Rogers, 1975) |
