# Supplementary material for: Changing behaviour ‘more or less’—do theories of behaviour inform strategies for implementation and de-implementation? A critical interpretive synthesis
Source: Implement Sci. 2018 Oct 29;13:134. doi: 10.1186/s13012-018-0826-6 (PMC6206907; doi:10.1186/s13012-018-0826-6)
Supplement: Supplementary file 3 — Theory descriptions reported to theorise strategies for changing frequency of behaviour. (DOCX 36 kb) [file 13012_2018_826_MOESM3_ESM.docx]

Additional file 3: Theory Descriptions reported to Theorise Strategies for Changing Frequency of Behaviour

| Theory | Description of Theory | Hypothesized impact on behaviour and behaviour change |
| --- | --- | --- |
| Control Theory (Carver & Scheier, 1990) | Behaviours are determined by:  A negative feedback loop whereby by individual’s perceptions of current behaviour/output are compared to a goal. Individual perceives their current behaviour/goal via an *input function,* and compares it to a *comparator***,** which is a particular standard. If there is a difference between the comparator and the behaviour then the individual will attempt to reduce discrepancy by performing the behaviour *(output function).* The behaviour has *an impact on the environment*, which changes the individual’s perception and the loop continues. | The goal is to change behaviour to reduce the perceived discrepancy between current state and the goal state. |
| Deterrent Theory (Blumstein et al., 1978) | Behaviour can be changed through the use of punishment and consists of  General deterrence, in which individuals receive punishment publically and are used as examples to deter others in the general population to act negatively in the future;  Specific deterrence whereby the individual is the focus and punishment is used to discourage the individual for acting negatively again. | Individuals will choose to commit or not commit crimes or undesired behaviour is based on the *certainty*, *swiftness*, and *severity* of the punishments. |
| Disconnected Values Model  (Anshel & Kang, 2007) | Behaviours are determined by  Positive habitual behaviours reflect the individual’s deepest values and will continue because the values support the behaviour.  Continuation of negative habitual behaviours reflects a disconnect from, or lack of awareness, about the individual’s own deepest values. | Identifying the ‘disconnect’ between the individual’s deepest values and beliefs and the negative behaviour is key to the model and the individual’s realization that the negative behaviour requires replacing with a *positive ritual.* |
| Goal Setting Theory (Locke & Latham, 1994) | Behaviour is determined by the setting of *goals* (defined as the object or aim of action) or intentions that the individual aspires to achieve. These goals affect task performance and can be moderated by such factors as *level of commitment, the importance of the goal, levels of self-efficacy, feedback, and complexity of task***.** | Goals that the individual sees as high priority, easy to complete and in which the individual receive some satisfaction in completion will likely result in the individual performing the behaviour to attain that goal. |
| Health Action Process Approach (Schwarzer et al., 2008) | Behaviour is determined by  1) Motivation phase (pre-intentional in which strength of intention (measured by *risk perception*, *outcome expectancies*, and *self-efficacy);*  2) *Volition* phase (self-regulatory or action phase focused on the cognitions involved in initiating and controlling the action | Addressing increasing behaviour - motivation phase is considered to be a causal process (i.e. individuals who have a higher risk perception will develop favourable outcome expectancies, which will in turn result in a higher self-efficacy) that leads to the formation of action plans to turn intention into action (Action Phase). |
| Health Belief Model (Rosenstock, 1974) | Behaviour is determined by individuals perceived health beliefs  Constructs include  Perceived illness threat (perceived susceptibility to the illness and perceived severity to, or consequences of, the illness), and  Evaluations of behaviours to counteract the threat, comprising perceived benefits weighed against perceived costs or barriers of performing the behaviour.  Internal and external triggers, or cues to action and health motivation (the value one places on one’s own health) can influence performance of an action. | If the threat of illness is perceived strong and benefits of performing the behaviour are greater than the barriers, individuals will more likely perform the necessary action to reduce the illness threat.  If health motivation is low, the behaviour may not be performed. |
| Implementation Intentions (Gollwitzer, 1996; Gollwitzer, 1999) | Behaviour can be changed through:  - Explicit plans an individual makes about when and where a behaviour (goal intention) will be achieved | By creating an implementation intention, individuals rely on external cues from the environment to trigger their behaviour. The more detailed the plan the more likely the behaviour will occur. |
| Operant Learning Theory (Skinner, 2005) | Behaviour can be changed through  Reinforcements - positive consequences that results in repetition of behaviour  Positive (addition of appetitive stimulus following correct behaviour)  Negative (removal of noxious stimulus once behaviour is performed or behaviour avoids noxious stimulus).  Punishment - unpleasant consequences that result in less frequent behaviour become less frequent  Positive (addition of noxious stimuli following inappropriate behaviour)  Negative (removal of appetitive stimuli following inappropriate behaviour)(Skinner, 2005).  Factors to consider:  *Scheduling* of the stimuli (either appetitive or noxious) refers to the rules of timing that controls when the stimulus is applied following behavioural response. They stimulus can be applied every time the behaviour is performed (continuous reinforcement), it can be applied after a period of time has passed (interval reinforcement), or after the behaviour has been performed number of times (ratio reinforcement).  Environmental *cues,* - individuals recognise to perform (or not perform) the behaviour, may also influence how effective reinforcement and punishment are at changing the frequency of behaviour. | Individuals will respond by increasing their behaviour to receive the reinforcement or will decrease the occurrence of the behaviour to avoid the punishment. |
| Protection Motivation Theory (Rogers, 1975; Rogers & Prentice-Dunn, 1997) | Behaviour is determined by  Protection intention (i.e. the intention to perform the recommended behaviour). Protection intention results from two appraisal processes:  Threat appraisal, which focuses on the threat and factors that increase or decrease the likelihood of the maladaptive response; weighs the intrinsic and extrinsic *rewards* of performing the maladaptive behaviour with the *severity* and *vulnerability* of the threat of the maladaptive behaviour  Coping appraisal, which reflects the coping responses of the individual in dealing with the threat and those factors that increase or decrease the probability of the adaptive response; reflects the belief that desired behaviour will be effective in reducing the threat (*response efficacy*) and the belief that the individual is capable of performing that behaviour (*self-efficacy*). | Depending on the strength of threat appraisal versus Coping appraisal, individual’s protecting intention will determine whether to perform the recommended behaviour or not. |
| Self Affirmation Theory (Steele, 1988) | Behaviour is change through the reflection upon an individuals values or strengths. Individuals are strongly motivated to maintain their sense of being a rounded, worthwhile person.  At the core of the theory is the idea of *self-integrity* (sense of being ‘‘competent, good, coherent, unitary, stable, capable of free choice, capable of controlling important outcomes’’), Individuals are highly vigilant to threats to their self-integrity and take steps to prevent it being damaged and to repair it when it is. | Individuals resist relevant health-risk information is because of the threat it presents to their self-integrity - messages suggesting they may need to change behaviour challenge the core notion of being competent, able, worthy. Resisting the message means less readiness to accept the information and to change behavior to reduce risk. |
| Self-Determination Theory (Deci & Ryan, 2008) | Behaviour is determined by the fulfilment of three basic needs:   1. *Competence:* the need to feel competent 2. *Autonomy*: the universal urge to be causal agents of one's own life and have choice 3. *Relatedness:* the want to interact, be connected to, and experience caring for others.   The type of motivation that drives an individual’s behaviour is more important than the amount of motivation they have. Intrinsic motivation (motivation due to inherent interest or enjoyment that promote behaviour) versus extrinsic motivation (comes from external sources and is divided into 4 types: Externally regulated behaviour, Introjected regulation of behaviour, Regulation through identification, Integrated Regulation. Intrinsic motivation is perceived to be a better motivator than extrinsic and the two can often impede the other from changing behaviour. | By focusing on the three psychological needs and improving intrinsic motivation behaviour can occur to achieve these needs. |
| Social Cognitive Theory (Bandura, 1977) | Behaviour is determined by  *Self-efficacy* (belief about one’s capability to perform an action to achieve a desired outcome (goal).  *Goals* are defined as self-incentives one has that guide the individual to perform the behaviour.  *Outcome expectancies* are the individual’s beliefs about possible consequence of their actions, conceptualised in two categories:  Situational outcomes – beliefs about which consequences will occur without individual action; and  Action-outcome – belief that a behaviour will or will not lead to a specific outcome. | Increasing an individual’s self-efficacy has the greatest impact on performance of the desired behaviour. |
| Temporal Self-Regulation Theory  (Hall & Fong, 2007) | Maladaptive behaviours are determined by a complex interaction between cognitive, biological and social factors. *Temporal proximity* and *value of anticipated benefits* as well as the *cost and outcomes of behaviour* determine whether behaviour will be performed. Determinants of behaviour are grouped in two categories: Motivational sphere and Ambient temporal contingencies.  Behaviours judged to be maladaptive in the long run, are driven by a strongly favourable balance of immediate costs and benefits. In contrast, many avoided behaviours that seem ‘‘adaptive’’ to the outside observer, are in fact associated with substantial costs (and few benefits) at the time of action. | By focusing on the benefits of the long-term outcomes rather than the immediate outcomes, individuals are more likely to perform the adaptive behaviour. In addition, if the behaviour is deemed successful, feedback loops may alter the temporal valuation and intentions are strengthen in such a way that the individual will likely perform the behaviour in the future. |
| Theory of Planned Behaviour (Ajzen, 1991) | Behaviour is determined by strength of *intention* to perform a behaviour, and *Perceived Behavioural Control* (the degree of perceived control over that behaviour)  Intention is determined by three variables:  *Attitude* towards the behaviour (a product of beliefs about its consequences and evaluations of those consequences),  *Subjective norm* (a product of perceptions of the views of other individuals or groups about the behaviour, and the strength of the individual’s desire to gain approval of these groups) and  *Perceived Behavioural Control* (a function of beliefs about factors likely to facilitate or inhibit the behaviour). | Targeting an individual’s intention through increasing positive attitudinal beliefs and subjective norms about the desired behaviour as well as increasing perceived control may be effective ways to increase the behavioural frequency. |
| Theory of Reasoned Action | Behaviour is determined by the strength of *intention* to perform a behaviour.  *Intention* is determined by three variables:  *Attitude* towards the behaviour (a product of beliefs about its consequences and evaluations of those consequences),  *Subjective norm* (a product of perceptions of the views of other individuals or groups about the behaviour, and the strength of the individual’s desire to gain approval of these groups) and | Targeting an individual’s intention through increasing positive attitudinal beliefs and subjective norms about the desired behaviour may be effective ways to increase the behavioural frequency. |
